# Characterization of Mg^2+^-regulated TRPM7-like current in human atrial myocytes

**DOI:** 10.1186/1423-0127-19-75

**Published:** 2012-08-14

**Authors:** Regina Macianskiene, Irma Martisiene, Danguole Zablockaite, Vida Gendviliene

**Affiliations:** 1 Institute of Cardiology, Lithuanian University of Health Sciences, Sukileliu 17, LT-50009, Kaunas, Lithuania

**Keywords:** Atria, Human, Myocyte, Mg^2+^, TRPM7

## Abstract

**Background:**

TRPM7 (**T**ransient **R**eceptor **P**otential of the **M**elastatin subfamily) proteins are highly expressed in the heart, however, electrophysiological studies, demonstrating and characterizing these channels in human cardiomyocytes, are missing.

**Methods:**

We have used the patch clamp technique to characterize the biophysical properties of TRPM7 channel in human myocytes isolated from right atria small chunks obtained from 116 patients in sinus rhythm during coronary artery and valvular surgery. Under whole-cell voltage-clamp, with Ca^2+^ and K^+^ channels blocked, currents were generated by symmetrical voltage ramp commands to potentials between -120 and +80 mV, from a holding potential of -80 mV.

**Results:**

We demonstrate that activated native current has dual control by intracellular Mg^2+^ (free-Mg^2+^ or ATP-bound form), and shows up- or down-regulation by its low or high levels, respectively, displaying outward rectification in physiological extracellular medium. High extracellular Mg^2+^ and Ca^2+^ block the outward current, while Gd^3+^, SpM^4+^, 2-APB, and carvacrol inhibit both (inward and outward) currents. Besides, divalents also permeate the channel, and the efficacy sequence, at 20 mM, was Mg^2+^>Ni^2+^>Ca^2+^>Ba^2+^>Cd^2+^ for decreasing outward and Ni^2+^>Mg^2+^>Ba^2+^≥Ca^2+^>Cd^2+^ for increasing inward currents. The defined current bears many characteristics of heterologously expressed or native TRPM7 current, and allowed us to propose that current under study is TRPM7-like. However, the time of beginning and time to peak as well steady state magnitude (range from 1.21 to 11.63 pA/pF, n_cells/patients_ = 136/77) of induced TRPM7-like current in atrial myocytes from different patients showed a large variability, while from the same sample of human atria all these parameters were very homogenous. We present new information that TRPM7-like current in human myocytes is less sensitive to Mg^2+^. In addition, in some myocytes (from 24 out of 77 patients) that current was already up-regulated at membrane rupture.

**Conclusions:**

This study provides the first electrophysiological description of TRPM7-like current in native human atrial myocytes. Less sensitivity to intracellular Mg^2+^ suggests for channel operation under physiological conditions. The TRPM7-like current up-regulation indicates the pathophysiological evidence of that current in human heart.

## Background

Within the past decade, TRPM7 (**T**ransient **R**eceptor **P**otential of the **M**elastatin subfamily) channels [1] (also named MagNuM, for **Mag**nesium **Nu**cleotide-regulated **M**etal ion channel [2], or MIC, for **M**agnesium **I**nhibited **C**hannel [3]) have been detected in a large number of tissues, including heart, using molecular approaches [2,4,5]. TRPM7 channels are important in human physiology, since they have been implicated in the regulation of transmembrane movement of Mg^2+^ in the cell [2,4,6-8] and of the cellular Mg^2+^ homeostasis [9,10]. Moreover, TRPM7 is required for cell viability [1,3,9], since both knockout and/or overexpression of these channels cause loss of cell adhesion, growth arrest, and rapid cell death [2,9,11]. Certain features, including the implication in the entry of extracellular Mg^2+^ (Mg_o_^2+^) and other divalent cations [12,13], as well as a bifunctional property with ion channel and kinase activities [2,4,6] discriminate TRPM7 from a variety of other known ion channels [7,8,14,15]. These channels are constitutively open [2]. However, under the physiological conditions they pass very weak inward current due to substantial down-regulation by high levels of intracellular Mg^2+^ (Mg_i_^2+^) and MgATP. By contraries, decreased Mg_i_^2+^ (in free or ATP-bound form) up-regulates the current carried by TRPM7 channels [2,8,15,16].

Data from different groups suggest that the altered function and/or regulation of TRPM7 channels could be implicated in pathological conditions such as anoxic cell death and ischaemia-reperfusion injury [5], arrhythmias [17], atria fibrillation [18], etc. However, it is important to note that TRPM7 channels in native cells have been detected mainly by using molecular biology and immunostaining methods [4,6]. The majority of electrophysiological studies so far were carried out either on heterologously expressed or on native RBL (rat basophilic leukemia cells), human Jurkat T-cells, and fibroblasts [2,4,7,12,16,18]. Although TRPM7 proteins are highly expressed in the heart [4,6], only few studies have been performed to detect or characterize the channels in native cardiomyocytes [15,19,20]. A non-selective channel, which is blocked by extracellular divalent cations [21] and inhibited by intracellular Mg^2+^[15], has been characterized in rat and pig ventricular myocytes [15,19,20]. Despite this indirect evidence, there are no reports, to date, on TRPM7-like current in human cardiomyocytes. Therefore, the aim of current study was designed to demonstrate the presence of TRPM7-like current and to characterize its biophysical and pharmacological properties in human atrial myocytes. Preliminary data have been presented as an abstract [22].

## Methods

### Patients

The study was carried out in accordance to the European Community guiding principles outlined in the declaration of Helsinki, with approval by the Ethics Committee of Biomedical Research of Kaunas Region, Lithuania (Nr.BE-2-60, 2009) and by the local Committee at the Hospital of Lithuanian University of Health Sciences.

The specimens of right atria were obtained during open-heart surgery from 116 patients of both sexes (mean age: 65.6 ± 1.02 years; age range: 40–81 years), all in sinus rhythm (SR), at the Hospital of Lithuanian University of Health Sciences. Only those patients in SR with no documented episodes of atrial fibrillation (AF) prior to surgery were included. Information on cardiac rhythm and rate was assessed using the pre-operative ECG. Most of them were treated with Ca^2+^-channel blockers, ACE-inhibitors, digitalis, diuretics, which were stopped 24 h before surgery. In addition, all patients received anaesthesia and antibiotics. Written informed consent before surgical procedures was obtained. The more detailed patients’ characters are presented in Table 1.

**Table 1:** Patients’ preoperative clinical characteristics

**Characteristic**	**Total n = 116; n and (%)**	**free-Mg**^**2+**^_**i**_**(all) n = 77; n and (%)**	**free-Mg**^**2+**^_**i**_**Without up-regulated TRPM7 at rupture n = 53; n and (%)**	**free-Mg**^**2+**^_**i**_**With up-regulated TRPM7 at rupture n = 24; n and (%)**
Age (years)	65.6±1.02	65.7±1.09	65.3±1.43	66.3±1.59
Gender (male/female)	73/43	49/28	30/23	19/5
Ischaemic heart disease	98(84.5)	68(88.3)	45(84.9)	23(95.8)
Myocardial infarction	47(40.5)	37(48.1)	25(47.2)	12(50.0)
Hypertension	97(83.6)	67(87.0)	43(81.1)	24(100.0)
Heart failure	54(46.6)	36(46.8)	21(39.6)	15(62.5)
Diabetes mellitus	15(12.9)	9(11.7)	0(0.0)	9(37.5)
Rheumatic heart disease	6(5.2)	6(7.8)	5(9.4)	1(4.2)
Type of heart surgery:				
coronary bypasses	63(54.3)	41(53.2)	32(60.4)	9(37.5)
valve repair/replacement	28(24.1)	16(20.8)	10(18.9)	6(18.9)
both (valves and bypasses)	25(21.6)	20(26.0)	11(20.8)	9(37.5)

Values are given as numbers of patients (n and %) with indicated clinical characteristics, except for age (MEAN±S.E.M.) and gender (numbers of male/female).

### Human atrial myocytes

The dissociation of the cells from small atrial specimen was performed immediately after surgery. The tissue was fine-cut in an oxygenated nominally Ca^2+^-free Tyrode solution (in mM: 135 NaCl, 5.4 KCl, 0.9 MgCl_2_, 0.33 NaH_2_PO_4_, 10 HEPES, and 10 glucose; pH 7.4 with NaOH) supplemented with 3 mg/ml BDM (2,3-butanedionmonoxime), which was washed out before enzyme application. The chunks were transferred to a beaker, which was placed into a water bath maintained at 37°C, and shaken with the Ca^2+^-free Tyrode solution containing 1 mg/ml BSA (Albumin, from bovine serum; SIGMA), 1 mg/ml collagenase (351 U/mg, type 2; Worthington), and 0.3 mg/ml protease (13 U/mg, type XXIV; SIGMA) by continuous bubbling with 100% O_2_. After 30 minutes, the solution with enzymes was replaced by fresh enzymatic solution containing only collagenase (1 mg/ml), and shaken until myocytes appeared. A small sample of supernatant was examined under the microscope every 5 minutes to determine the number and the quality of the isolated myocytes. When the yield appeared to be maximal, the chunks were resuspended in a Ca^2+^-free Tyrode solution and gently pipetted. The resulting suspension was centrifuged, and myocytes were stored at room temperature in Tyrode solution with nominally zero Ca_i_^2+^ in order to calm down (for about 15–20 min). Afterward, special attention was given to the period of adjustment to low-calcium containing solutions to protect myocytes from the damage by extracellular Ca^2+^ ions. Therefore, Ca^2+^ concentration was raised gradually to 2, 5, 10, and 18 μM. Thus, nominally Ca^2+^-free concentration (due to trace amounts of Ca^2+^ in enzymes, glucose, and other reagents), lower temperature (room), and progressive attune to extracellular Ca^2+^ helped from irreversible myocyte damage. In addition, extracellular Mg^2+^, which was constantly kept in all isolation solutions, protects from sustained Na^+^ entry through the divalent ions-selective channels. All those factors allowed to reduce partially the “calcium paradox” phenomenon during myocyte isolation. Overall, the major contributors that influenced the limited yield, which varied from 5 to 20%, of atrial myocytes after isolation were: age of the patient, the level of fibrosis (due to cardiac pathologies), high level of adipose tissue, and viability of the cells survived on Ca^2+^ readmission procedure. Only Ca^2+^-tolerant, a rod-shaped, with clear striation, and without sarcolemmal blebs myocytes were selected for the recordings. All experiments were performed at room temperature (+21 ± 2°C).

### Electrophysiology

Whole cell currents were recorded using a VP-500 patch-clamp amplifier (BioLogic, France), data digitised at 5 kHz, and filtered at 1 kHz. Patch electrodes were pulled to a resistance of about 1.5 MΩ. In all experiments extracellular solutions were applied using a rapid solution changer (BioLogic, France). Cells were depolarised from a holding potential of -80 mV. Currents were measured using 4 s long symmetrical voltage ramps from -120 mV to +80 mV and then back to -120 mV, applied every 10 s. The protocols were designed to block voltage-dependent channels, i.e. the ascending limb allowed for activation and subsequent inactivation of Na^+^ and T-type Ca^2+^-currents. Currents were recorded during the descending limb while the voltage-dependent currents were still inactivated. To dissociate the current under study from non-channel leak currents, two criteria were strictly applied before considering a current change as reflecting a genuine membrane conductance change: 1) the change had to develop progressively and smoothly (without large current jumps) over time; 2) the increase in conductance had to be reversible upon application of physiological concentration of extracellular Ca^2+^ and Mg^2+^.

### Solutions and drugs

All solutions were designed to block K^+^ (K^+^ replaced by Cs^+^) and L-type Ca^2+^-channels (10 μM nifedipine added). The external Tyrode solution contained (in mM): 135 NaCl, 5.4 CsCl, 0.9 MgCl_2_, 1.8 CaCl_2_, 0.33 NaH_2_PO_4_, 10 HEPES, and 10 glucose (pH 7.4; with NaOH). Extracellular divalent-free (DVF) solutions were prepared by omitting Ca^2+^ and Mg^2+^ cations. Na^+^-free solution prepared by replacing with nonpermeable NMDG^+^ (N-methyl-D-glucamine), which was reduced to maintain osmolarity in experiments to study divalent cation permeability. The composition of the internal solution was (in mM): 130 Cs-glutamate, 25 CsCl, 5 Na_2_ATP, 1 EGTA, 0.1 Na_2_GTP, and 5 HEPES (pH 7.25; with CsOH). Internal solutions were modified by omitting or adding different concentrations of MgCl_2_ or MgATP, and by substituting EGTA with EDTA. The free Mg^2+^ concentration was calculated using CaBuf software (ftp://ftp.cc.kuleuven.ac.be/pub/droogmans/cabuf.zip).

All reagents were from Sigma-Aldrich, and were prepared as stock solutions (100 mM): spermine (SpM^4+^) and Gd^3+^ (chloride salt) in distilled water; 2-APB (2-aminoethoxydiphenyl borate) in dimethyl sulfoxide (DMSO); nifedipine and carvacrol in ethanol. The stock solutions were diluted to the desired concentrations. We have shown before that <0.1% level of DMSO and <0.01% of ethanol did not have any effect of its own.

### Statistics

Data are presented as MEAN ± S.E.M., with *n* indicating the number of cells/patients (n_c/p_) studied. Means were compared using analysis of variance (ANOVA) and Student’s *t* test. One-way ANOVA was used for assessing differences between data of different cells (i.e. without *vs.* with up-regulated current at rupture) or when the different concentrations of intracellular Mg^2+^ were applied. Paired Student’s *t* test was used for evaluating the difference between means of current magnitude at rupture *vs.* current up-regulation within time. P < 0.05 was considered statistically significant (*).

## Results

### Presence of TRPM7-like current in human atrial myocytes

**Figure 1:**
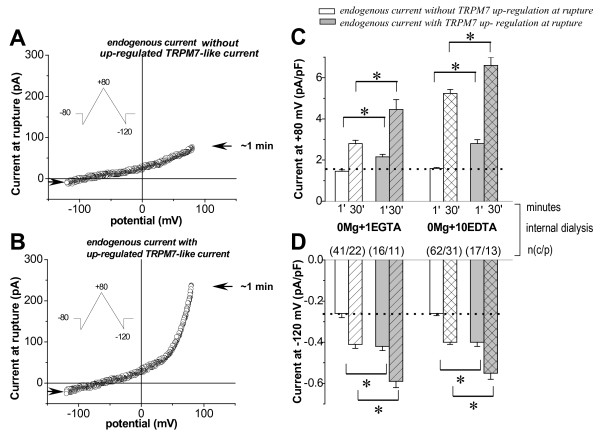
**Presence of TRPM7-like current in human atrial myocytes of patients with normal SR.** Original traces of endogenous currents without (**A**) and with already up**-**regulated TRPM7-like current (**B**, notice outward rectification) obtained using voltage ramps (see insert) at cell membrane rupture are plotted against voltage. The arrow indicates current peak value (at +80 mV and **-**120 mV) at rupture. Summary values (± S.E.M., and *n*-cells/patients) of endogenous (white) and already up-regulated (grey) currents: at rupture (~1 min; plane), after 30 min of dialysis with EGTA (striped), and EDTA (crossed) as indicated at +80 (**C**) and **-**120 mV (**D**). Dot line indicates endogenous current density at rupture. Differences between endogenous and already up-regulated currents at rupture as well as steady state values of TRPM7-like current were significant (*P <0.001) when paired *t* test and ANOVA were used.

**Figure 2:**
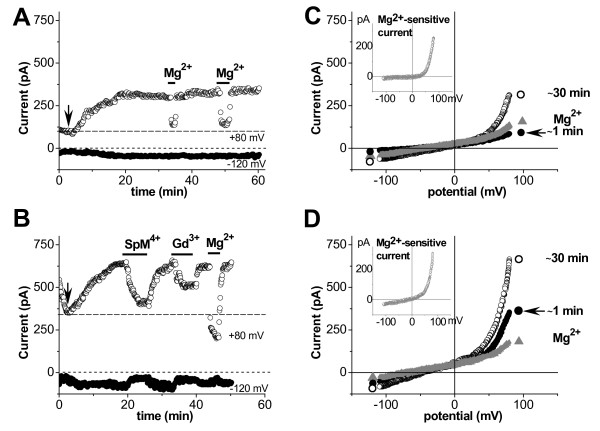
**Different sensitivity to high extracellular Mg**^**2+**^**.** Time diaries of currents from different myocytes, i.e. without- and with up-regulated TRPM7-like current at rupture (**A** and **B**, respectively), are plotted against time at +80 (ο) and -120 mV (), and external inhibition by 7.2 mM Mg^2+^, 100 μM SpM^4+^, and 100 μM Gd^3+^ (periods of exposure are indicated by horizontal bars). Corresponding I/V relations (**C, D**) obtained at rupture (), after 30 min of dialysis (○) and after external application of 7.2 mM Mg^2+^ (▴), respectively. The arrow and horizontal dashed line indicate current amplitude at rupture. Notice different sensitivity to high Mg_o_^2+^**.** In inserts – corresponding Mg^2+^-sensitive currents. Horizontal dot line - zero current level.

The TRPM7-like current induced by low levels of intracellular Mg^2+^ (Mg_i_^2+^) has been previously characterized in pig ventricular myocytes [19,20]. Here we examined whether the same current exists in freshly isolated human atrial myocytes of patients with normal SR. Because that current is up-regulated when Mg_i_^2+^ is low, first, we tested the effect of elimination Mg_i_^2+^ in the cell, while superfusing with physiological concentrations of extracellular Ca^2+^ (Ca_o_^2+^) and Mg^2+^ (Mg_o_^2+^). Figure 1 A and B show current–voltage (I/V) relationship curves of currents recorded at patch rupture in two separate atrial myocytes, dialyzed with zero Mg_i_^2+^. Obviously, in first myocyte, as demonstrated in Figure 1A, that current (endogenous) was relatively small and without outward rectification (see also Figure 2C, indicated by arrow). While in other, along with endogenous, the much large current with an outwardly rectifying I/V relation, as presented in Figure 1B, was obtained (see also Figure 2D, indicated by arrow). In cardiomyocytes, differently to heterologous expression system, the endogenously expressed current(s) other than TRPM7-like might exist. Despite the TRPM7 channels are constitutively active but under physiological conditions their activity is highly down-regulated [2]. Since in our study we measured the total of endogenously expressed currents, we believe that current in Figure 1A in a large part is non-TRPM7 contaminating though little of TRPM7-like currents, which, possibly, of down-regulation cannot be obtained electrophysiologically right at membrane rupture. That current further will be referred as the small endogenous without TRPM7 up-regulation at rupture. However, the much large current presented in Figure 1B may suggest for TRPM7-like current up-regulation to a higher extend, which could exceed the non-TRPM7 endogenous currents. In both atrial myocytes, as presented in Figure 1 (A and B), the reversal potential (E_rev_) is shifted to the negative potentials. This also points to the contamination of the whole cell currents by endogenously expressed other currents, which can be activated in parallel with the TRPM7-like current, and may have an impact on E_rev_. Despite Cs^+^-based internal and external solutions helped for various K^+^ channels elimination, nevertheless, at least one of them, such as rapid delayed rectifying K^+^ channel, is permeable to Cs^+^[23], and this could be the reason for such negative E_rev_ under experimental conditions used. The averaged current density of both type records at positive and negative potentials is presented in Figure 1C and D (plane white and grey columns on the left), respectively. According to our data, in the majority of the cells, when the membrane of atrial myocyte was ruptured, a small endogenous, without TRPM7-like current up-regulation, current (1.46 ± 0.07 pA/pF at +80 mV, and -0.27 ± 0.02 pA/pF at -120 mV, n_c/p_ = 41/22) persisted in the presence of blockers of classic (K^+^ and voltage-dependent Ca^2+^) channels. However, in part of human atrial myocytes, under the same experimental conditions, larger currents (2.16 ± 0.11 pA/pF and -0.42 ± 0.02 pA/pF, respectively, at +80 and -120 mV, n_c/p_ = 16/11) were obtained at cell membrane rupture. This unexpected result is unlike animal data and suggests the presence of already up-regulated TRPM7-like current in human atrial myocytes.

Nevertheless, in all myocytes, i.e. without and with up-regulated current at rupture, as shown in Figure 2A and B, with continued cell dialysis the currents gradually increased with time, and reached a plateau in 20–25 min. Mean data of TRPM7-like current steady-state values of both type records at positive (2.80 ± 0.16 pA/pF and 4.46 ± 0.48 pA/pF) and at negative potentials (-0.42 ± 0.02 pA/pF and -0.55 ± 0.03 pA/pF) are shown in Figure 1C and D (striped white and grey columns on the left), respectively. The increase of outward currents was by large more prominent than the increase of inward currents (notice different scale). The steady-state magnitude of the up-regulated current was stable for 60 min and longer. The I/V relationships obtained at rupture and after 30 min of dialysis, as well as the Mg^2+^-sensitive current (calculated as the difference in I/V relationships between the up-regulated and endogenous current; see inserts) with reversal potential close to zero mV are presented in Figure 2C and D.

We also performed experiments by using EDTA instead of EGTA in the internal solution, in order to increase the buffering of internal Mg_i_^2+^ and to yield maximal activation of TRPM7 channels. This resulted in a significant (P < 0.0001) increase in outward current amplitude in all atrial myocytes (without or with up-regulated current at rupture, respectively, from 1.60 ± 0.05 to 5.24 ± 0.19 pA/pF, n_c/p_ = 62/31, and from 2.80 ± 0.19 to 6.60 ± 0.53 pA/pF, n_c/p_ = 17/13; see Figure 1C, crossed white and grey columns on the right). However, the inward current component was not much changed by the increased intracellular Mg_i_^2+^ chelation (Figure 1D, crossed *vs.* striped columns). In addition, during cell dialysis with EDTA the outward current started to develop earlier (at 2.52 ± 0.18 min) and reached plateau faster (at 12.5 ± 0.46 min), as compared with those in which EGTA was used (current up-regulation started at 5.88 ± 0.44 min, and the plateau was achieved at 22.43 ± 1.67 min). Such acceleration of the current up-regulation in cells dialyzed with EDTA could be attributed to a faster and more pronounced decrease in the cytosolic free-Mg_i_^2+^ levels. In further experiments, the myocytes were dialysed with EDTA-containing internal solution.

Since the sensitivity of TRPM7 channels to Mg^2+^ is an essential condition, we examined whether the above described current is inhibited by high concentrations of extracellular Mg_o_^2+^. Figure 2 demonstrates that 7.2 mM of Mg_o_^2+^ effectively blocked the outward component of up-regulated current, while the inward component was almost unaffected, as it is expected for current carried by TRPM7 channels. We noticed that in cells with small endogenous current at rupture, high Mg_o_^2+^ reduced the outward current amplitude to levels close to those measured at cell rupture (Figure 2A and C). In contrast, in those cells with large current at rupture, possibly activated TRPM7-like, high Mg_o_^2+^ reduced the up-regulated current to lower levels (2.0 ± 0.15 pA/pF, n_c/p_ = 12/11) than those presented at the cell rupture (Figure 2B and D). The deeper blocking effect in cells with large currents at rupture is in support of the view that TRPM7-like currents were already up-regulated in these cells. The effect of high Mg_o_^2+^ was fully reversible and reproducible. Similar effect was obtained and by using Ca_o_^2+^ instead of Mg_o_^2+^ (not illustrated). The data above demonstrate a blocking effect of the extracellular divalent cations on the current under study.

**Figure 3:**
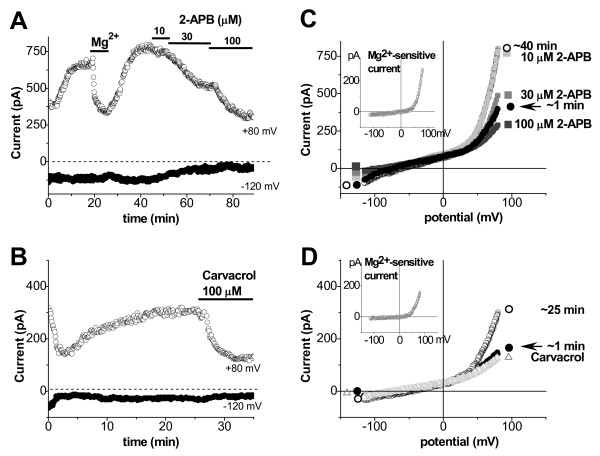
**TRPM7**-**like current inhibition by 2**-**APB and carvacrol.** Time course of changes in membrane currents measured at +80 mV (○) and -120 mV () during ramp depolarizations (**A** and **B**) and corresponding I/V relations (**C** and **D**) at rupture (indicated by arrow), after more than 20 min of dialysis (○), and after external application of 2-APB (■): 10 μM (light grey), 30 μM (grey), and 100 μM (dark grey), and 100 μM carvacrol (Δ), respectively.

Generally, the defined characteristics, e.g. the steep outward rectification, time independency during a voltage steps (not illustrated), reversal potential (of calculated Mg^2+^-sensitive current) close to zero mV (see Figure 2 and Figure 3 inserts in C and D), and inhibition by extracellular Mg_o_^2+^ and Ca_o_^2+^ make that current similar to the MagNuM/MIC [2,3,7,8] or TRPM7-like current [12,13,19]. However, the important new information is that TRPM7-like current, blocked by high Mg_o_^2+^, was already up-regulated at rupture in part of human atrial myocytes of patients with normal SR (24 out of 77).

### Inhibition by TRPM7 channel blockers

Further confirmation that TRPM7 channels carry current described above, could be obtained by testing TRPM7 channel inhibitors. Here we applied the following compounds, used to block these channels: Gd^3+^ (generally used to block various non-selective currents [21]), SpM^4+^ (which allows discriminating TRPM7 from store-operated channels [15]), and 2-APB (which assists separating TRPM7 from TRPM6 channels [13]). All of them at 100 μM concentration caused a significant (*P* <0.01) decrease of up-regulated current. Outward currents were decreased to 2.55 ± 0.37, 2.22 ± 0.22, and 1.72 ± 0.38 pA/pF by Gd^3+^ (n_c/p_ = 10/9), SpM^4+^ (n_c/p_ = 6/5), and 2-APB (n_c/p_ = 4/4), respectively, and the inward currents were decreased to -0.26 ± 0.03, -0.25 ± 0.03, and -0.29 ± 0.05 pA/pF, respectively (Figure 2B, and Figure 3A and C). In addition, we tested for the effect of carvacrol, which recently has been reported to suppress TRPM7 channels (at 500 μM) in HEK cells [24]. In our hands, 100 μM of carvacrol was needed for complete inhibition of outward (to 1.93 ± 0.31 pA/pF) and inward currents (to -0.34 ± 0.04 pA/pF) in low-Mg^2+^ dialyzed human atrial myocytes (n_c/p_ = 8/8) (Figure 3B and D). These results further indicate the possible involvement of TRPM7 channels.

### Inhibition by intracellular Mg^2+^

**Figure 4:**
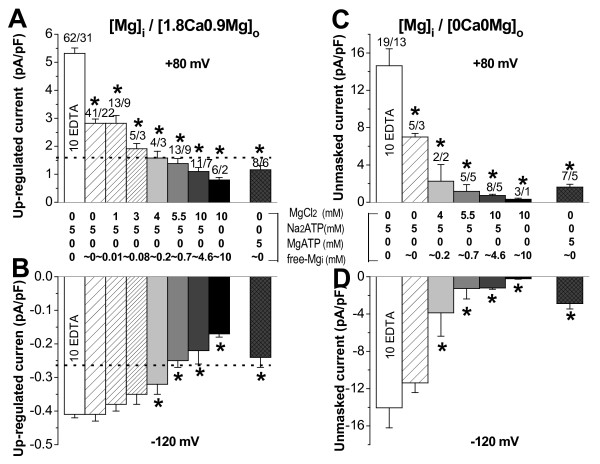
**TRPM7**-**like current down**-**regulation by intracellular Mg**^**2+**^**.** Mean data (± S.E.M., *n*-cells/patients) of up-regulated and additionally unmasked TRPM7-like outward (+80 mV) and inward (-120 mV) current amplitude, respectively, in the presence (**A** and **B**) or absence (**C** and **D**) of extracellular divalent cations, when different Mg_i_^2+^ in each dialysis solution was used, as indicated (in mM). Dot line indicates endogenous current density at rupture, which varied between 1.51 and 1.63 pA/pF at +80 mV, and -0.25 and -0.32 pA/pF at -120 mV. *P<0.001 *vs.* current value in Mg_i_^2+^-chelated myocytes when ANOVA was used.

Another main feature of TRPM7 channels is down-regulation of current by high Mg_i_^2+^ in its free or ATP-bound form. We measured currents during 40–50 min of cell dialysis with solutions containing the following free-Mg_i_^2+^ concentrations (in mM): ~0.014, ~0.078, ~0.174, ~0.715, and ~4.62 (calculated using CaBuf program, when the pipette solution contained 1 EGTA, 5 Na_2_ATP, and MgCl_2_ was added at 1, 3, 4, 5.5 or 10 mM, respectively). Adding of 10 mM MgCl_2_ to a similar internal solution, but from which Na_2_ATP was removed, allowed to obtain about 10 mM of free-Mg_i_^2+^. Figure 4A and B illustrate that increasing free-Mg_i_^2+^ in the dialysis solution resulted in a progressive down-regulation of currents. It is interesting to notice that no current up-regulation was obtained when the physiological (~0.72 mM) free-Mg_i_^2+^ was applied in the pipette. Instead, under these conditions, down-regulation of both outward and inward current components (to 1.38 ± 0.18 pA/pF and -0.25 ± 0.02 pA/pF, respectively) developed after prolonged dialysis (~80 min). This down-regulation by physiological free-Mg_i_^2+^ was incomplete, since high (7.2 mM) Mg_o_^2+^ was still able to reduce the outward current amplitude to 1.15 ± 0.2 pA/pF (n_c/p_ = 5/5; not illustrated). These results suggest that the TRPM7-like current is active under physiological free-Mg_i_^2+^ levels. The higher than physiological free-Mg_i_^2+^ (4.6 or 10 mM) caused a more pronounced down-regulation of both current components (see Figure 4A and B, dark grey and black columns).

Since, Mg_i_^2+^ in its bound form can also suppress TRPM7-like current, next, we dialyzed myocytes with internal solution containing 0 mM MgCl_2_ and 5 mM MgATP (free-Mg_i_^2+^ ~0). Under these conditions, the up-regulation of currents was markedly inhibited. After about 60 min of dialysis, steady-state outward currents at +80 mV and inward currents at −120 mV were 1.16 ± 0.15 pA/pF and −0.24 ± 0.03 pA/pF, respectively (Figure 4A and B, crossed dark grey columns). These results confirm the dual sensitivity of the channel under study to free-Mg_i_^2+^ and MgATP, as it is documented for TRPM7 channels [2,7,8,12,13,15,19].

### Unmasked TRPM7-like current in extracellular DVF solutions

**Figure 5:**
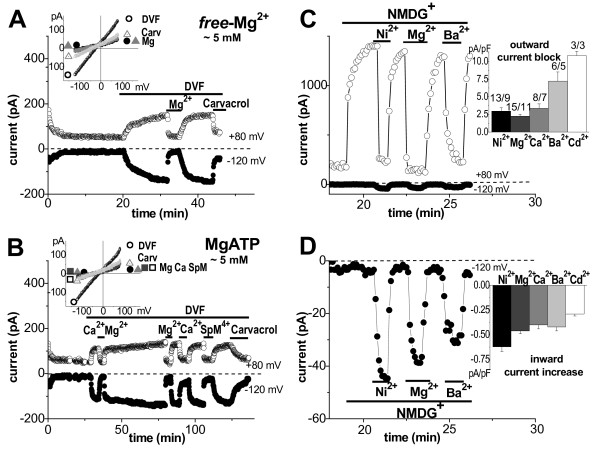
**TRPM7-like current regulation by extracellular divalent cations.** Unmasked current in extracellular DVF medium during dialysis with ~5 mM free-Mg_i_^2+^ (**A**) or 5 mM MgATP (**B**): at +80 (○) and −120 mV (). The DVF extracellular solution contained 100 μM of nifedipine, and Mg^2+^ (7.2 mM), Ca^2+^ (1.8 mM), SpM^4+^ (100 μM), or carvacrol (100 μM) added on top. In inserts – I/V relations: Cs^+^-Tyrode (), DVF (ο), Mg^2+^ (▴), Ca^2+^ (■), SpM^4+^ (□), or carvacrol (Δ). Permeability to divalent cations at +80 (**C**) and -120 mV (**D**), and mean data in inserts, respectively.

TRPM7 channels at physiological negative membrane potentials carry only very small inward currents in the presence of extracellular divalent cations. However, the inward current becomes larger and carried by monovalent cations in divalent-free (DVF) extracellular solution, because of a removal of channel block by Ca_o_^2+^ and Mg_o_^2+^[2,15,21]. Therefore, to further characterize the channels present in human atrial myocytes, we applied DVF extracellular solution on cells dialyzed with Mg^2+^-free intracellular solution. This resulted in a significant (P < 0.0001) increase of both outward and inward current amplitude (see mean data in Figure 4C and D, white columns). Further we verified, whether the large currents in DVF solutions can be modified by intracellular Mg_i_^2+^. Mean data, presented in Figure 4C and D, show that when higher Mg_i_^2+^ was used, smaller additional current was unmasked in DVF solutions. That additionally unmasked current was suppressed upon readmission of Mg_o_^2+^, Ca_o_^2+^ or TRPM7 channel blockers (Figure 5A and B). Under DVF conditions I/V relations were linearized, the reversal potential was close to 0 mV, as it is demonstrated in Figure 5A and B (inserts). We noticed, however, that quite big currents still could be unmasked in DVF solutions, when cells were dialysed with 5 mM of intracellular free- (Figure 5A) or bound-Mg_i_^2+^ (Figure 5B). The current was not due to the movement of monovalent ions via L-type Ca^2+^-channels [25] because it was resistant to 100 μM nifedipine, and because the difference current obtained on the ascending limb of the ramp (from a holding potential of -80 mV, which inactivated L-type Ca^2+^-channels) was practically the same as the one obtained on the descending limb (not illustrated).

### Permeability of TRPM7-like channel to divalent cations

Finally, divalent cations themselves can permeate TRPM7 channels [12,13,19]. We also tested the permeation of human cardiomyocytes to a range of divalent cations. In these experiments we used DVF internal/external solutions and, in addition, to prevent Na^+^ from passing along with divalent cations, extracellular Na^+^ was replaced by the non-permeable cation, NMDG^+^. This resulted in a large increase of outward, and a suppression of inward monovalent cation current. Upon switching to an NMDG^+^-based solution containing 20 mM of Mg_o_^2+^ or other divalents, a simultaneous decrease in outward and an increase in inward currents were obtained (Figure 5C and D, see average responses in inserts). These effects were reversible and reproducible. In our hands the largest increase in inward current was obtained with Ni^2+^, which is consistent with the results reported by others on heterologously expressed TRPM7 [12] or in pig ventricular myocytes [19], hence further strengthening the view that TRPM7 channels underlie the current measured in the present study. In human atrial myocytes the ion sequence profiles for divalent cations were Mg^2+^>Ni^2+^>Ca^2+^>Ba^2+^>Cd^2+^ for decreasing outward and Ni^2+^>Mg^2+^>Ba^2+^ ≥ Ca^2+^>Cd^2+^ for increasing inward currents.

## Discussion

In this study, we have demonstrated electrophysiologically the presence of TRPM7 channels in native, freshly isolated human atrial myocytes, and characterized the biophysical and pharmacological properties of TRPM7-like current, such as: 1) dual regulation by Mg_i_^2+^ (in free-Mg^2+^ or ATP-bound form), 2) up- and down-regulation by low and high levels of intracellular Mg^2+^, 3) steep outward rectification in the presence of divalent cations and linear I/V relations in DVF extracellular solutions, 4) reversal potential (of calculated Mg^2+^-sensitive current) of approximately 0 mV, 5) block by extracellular Mg_o_^2+^, as well as channel permeation (Ni^2+^>Mg^2+^>Ba^2+^≥Ca^2+^>Cd^2+^) by physiologically relevant Ca^2+^ and Mg^2+^ ions, and other non-physiological divalent cations, 6) inhibition by Gd^3+^, SpM^4+^, 2-APB or carvacrol. Despite in native cardiomyocytes the magnitude of Mg^2+^-regulated current as well E_rev_ under physiological extracellular conditions could be obtained as the difference between the steady-state value of the induced current up-regulation (which is due to magnesium washout during cell dialysis with Mg^2+^-free internal solution) and the current magnitude at cell membrane rupture (which consist of both small TRPM7-like and other(s) endogenously expressed non-TRPM7 currents), the overall data attained in human atrial myocytes, in many respects, is similar to that of TRPM7/MagNUM/MIC [2-8,12,13,15,19], and allowed us to propose that the current described here is TRPM7-like. However, diversely to other cell types (heterologously expressed or native RBL, human Jurkat T-cells), there was a large variability in the magnitude of the TRPM7-like current (range from 1.21 to 11.63 pA/pF, n_c/p_ = 136/77). Besides, the time of beginning (from 1 to 20 min) of current up-regulation and time to peak (from 10 to 40 min) were variable as well, and it might be associated with the different clinical history of the patients. This notwithstanding, from the same sample of human atria all these parameters were very homogenous, and data obtained from 2–5 distinct cardiomyocytes were similarly sized. This allowed us to assume that they are characteristic for the given patient.

Here we present new information that TRPM7-like current in human myocytes is less sensitive to Mg^2+^, since substantial current can still be obtained with physiological intracellular Mg_i_^2+^. This implies a possible operation of the channel under physiological conditions. In addition, important and unexpected new result of the present study is that the TRPM7 channels could be already up-regulated at cell membrane rupture, and carry substantial outward as well inward currents in isolated atrial myocytes obtained from biopsies of human heart with normal SR (in 24 out of 77 patients). Such current up-regulation right at patch rupture was never obtained in cardiomyocytes from animal hearts [15,19,20]. The underlying differences might be of species-specifics, and because TRPM7-like current mostly was studied on relatively healthy and young animals. The results of this study are probably influenced by many confounding factors, e.g. age, medication, and concurrent disease (underlying ischaemic cardiac disease, hypertension, heart failure, diabetes mellitus, etc.) (see Methods, Table 1).

TRPM7 is highly expressed in the heart, and the density in fibroblasts, atrial and sinoatrial cells is higher than in ventricular cardiomyocytes, however, the electrophysiological characterization in their native cellular environment is limited [15,18-20]. Such data on human cardiac cells, to date, are even more scant. In fact, using electrophysiology methods, only in human atria fibroblasts TRPM7-like current has been described [18], and was demonstrated its deleterious role during AF. We used atrial myocytes, and of patients with normal SR, therefore, already up-regulated current at rupture is likely related to the patient’s clinical history other than AF.

Numerous studies indicate that pathologies such as type II diabetes [26], hypertension [27], heart failure [28], and arrhythmias [17] are associated with lower levels of Mg_i_^2+^. Consequently, the magnitude of TRPM7-like current in human cardiomyocytes might be enhanced under these pathologies. Besides, the activation of the TRPM7-like current in the heart might be altered by factors such as acidosis [13,19] or metabolic depletion [29], that are major components of acute myocardial ischaemia. On the other hand, a depressed level of Mg_i_^2+^ can itself cause significant cardiomyocytes dysfunction in the absence of any cardiac disease as it was demonstrated on rat cardiac myocytes [30]. Since, the intracellular Mg^2+^ is a key component for all enzymatic reactions that require ATP and kinases, therefore, even small changes in Mg^2+^ uptake may exert cation derangement with abnormalities in intracellular Ca^2+^ homeostasis. Till now, the TRPM7-dependent pathways that are affected in various pathologies in human heart remain not clear. Therefore, future work is needed to discriminate and clarify the conditions, which would provoke or favour the up-regulation of TRPM7-mediated currents in human cardiomyocytes.

Now it is evident the physiological role of TRPM7/MagNUM/MIC current to maintain cellular Mg^2+^ homeostasis [9,10]. Data from different groups suggest that under physiological conditions the TRPM7 channel is involved in Mg^2+^ influx into the cell [12], and that it may play a role in cell growth and proliferation [9-11,31]. In addition to Mg^2+^, TRPM7 also transports into the cell Ca^2+^ cations and other physiological and toxic metals, which permeate the channel even in the presence of physiological levels of Ca_o_^2+^ and Mg_o_^2+^[12]. A model [32] for ion permeation through these channels suggests that under physiological conditions the ability of Mg^2+^ to enter the channel is higher versus permeability to Ca^2+^ cations, while under reduced extracellular Mg^2+^ concentration, the ability of Ca^2+^ cations to enter the channel is increased. The enhanced Ca^2+^ entry into the cell via TRPM7 channel could induce the intracellular Ca^2+^ overload (clinical Ca^2+^ paradox), which may lead to various heart pathologies.

Till now, the physiological/pathophysiological functions of TRPM7 channels in the heart have been derived from numerous studies on recombinant expression systems. In human cardiomyocytes the TRPM7-like current, described above, was up-regulated at rupture by as yet unclear disease condition, as to now, we are unable to offer plausible explanations for this. Unless, current data indicate that it might be related with the increased severity of disease (see Table 1). Besides, the relationship between induced TRPM7-like current and alterations of the electrical properties of human heart are not determined yet, and this will be the focus of our future work. A better understanding of the role of TRPM7 channels in their native environment will provide important clues on the mechanisms underlying various heart diseases.

## Conclusions

The overall results of our study have demonstrated electrophysiologically for the first time the presence of intracellular/extracellular Mg^2+^-regulated current, resembling TRPM7/MagNUM/MIC, in freshly isolated human atrial myocytes. The channel may play a role in cardiac ion homeostasis, especially involving Mg^2+^ and Ca^2+^ permeation, and operate under physiological conditions. In addition, we describe an unexpected novel result that the TRPM7-like channels are already up-regulated in part of human atrial myocytes, from patients with normal SR, therefore, might be important under certain pathological situations.

## Competing interests

The authors declare that they have no competing interests.

## Authors’ contributions

RM designed the experiments. All authors performed experiments. IM and DZ analysed data/statistics. RM and VG discussed the content. RM wrote the paper. All authors discussed the results, commented on the manuscript, and approved the final version before submission.
